# Assessment of postoperative adjuvant treatment using toceranib phosphate against adenocarcinoma in dogs

**DOI:** 10.1111/jvim.15768

**Published:** 2020-04-08

**Authors:** Hiroki Yamazaki, Toshiyuki Tanaka, Keiichiro Mie, Hidetaka Nishida, Naoki Miura, Hideo Akiyoshi

**Affiliations:** ^1^ Veterinary Medical Center, Graduate School of Life and Environmental Sciences Osaka Prefecture University Osaka Japan; ^2^ Veterinary Surgery, Graduate School of Life and Environmental Sciences Osaka Prefecture University 1‐58 Rinku‐oraikita, Izumisano, Osaka Japan; ^3^ Veterinary Teaching Hospital, Joint Faculty of Veterinary Medicine Kagoshima University Korimoto, Kagoshima Japan

**Keywords:** adenocarcinoma, postoperative adjuvant treatment, toceranib phosphate, tumor microenvironment

## Abstract

**Background:**

Toceranib phosphate (TOC) could be made widely available for treating tumors in dogs if evidence shows that TOC inhibits recurrence after surgery.

**Objectives:**

To investigate how postoperative adjuvant treatment with TOC modulates the tumor microenvironment (TME), by assessing effects on angiogenic activity, tumor‐infiltrating regulatory T cells (Tregs), and intratumoral hypoxia.

**Animals:**

Ninety‐two client‐owned dogs were included: 28 with apocrine gland anal sac adenocarcinoma, 24 with small intestinal adenocarcinoma, 22 with lung adenocarcinoma, and 18 with renal cell carcinoma.

**Methods:**

Retrospective, multicenter study comparing time to progression (TTP) between 42 dogs treated by surgery and TOC and 50 dogs treated by surgery alone. Differences were analyzed in the expression of vascular endothelial growth factor receptor‐2 (VEGFR2) and the number of Foxp3^+^ Tregs and hypoxia‐inducible factor (HIF)‐1α^+^ cells in tumor tissues sampled at the first and second (recurrence) surgeries.

**Results:**

Median TTP for dogs treated by surgery and TOC (360 days) was higher than that for dogs treated by surgery alone (298 days; hazard ratio, 0.82; 95% confidence interval [CI], 0.65‐0.96; *P* = .02). In dogs treated by surgery and TOC, VEGFR2 expression and the number of Tregs and HIF‐1α^+^ cells were significantly lower in tissues sampled at the second surgery than in those sampled after the first surgery. In dogs treated by surgery alone, significant differences were found between samples from the 2 surgeries.

**Conclusions and Clinical Importance:**

Toceranib phosphate could prove to be a useful postoperative adjuvant treatment because of its modulation of the TME.

**Abbreviations**
AEs: adverse events
AGASA: apocrine gland anal sac adenocarcinoma
CI: confidence intervals
HIF: hypoxia‐inducible factor
HPFs: high power fields
HR: hazard ratio
KIT: KIT proto‐oncogene receptor tyrosine kinase
LA: lung adenocarcinoma
PDGFR: platelet‐derived growth factor receptor
RCC: renal cell carcinoma
SBA: small bowel adenocarcinoma
TKI: tyrosine kinase inhibitor
TME: tumor microenvironment
TOC: toceranib phosphate
Tregs: regulatory T‐cells
TTP: time to progression
VEGFR2: vascular endothelial growth factor receptor‐2

## 1 INTRODUCTION

Toceranib phosphate (TOC) is a multitarget tyrosine kinase inhibitor (TKI), that exerts antiangiogenic and antitumor effects by targeting molecules such as platelet‐derived growth factor receptor (PDGFR), vascular endothelial growth factor receptor‐2 (VEGFR2), and KIT proto‐oncogene receptor tyrosine kinase (KIT).^1^, ^2^ It has shown clinical utility as a single agent in dogs with mast cell tumors, gastrointestinal stromal tumors, and some carcinomas.^3^, ^4^ In 1 study, inhibition of PDGFR, VEGFR2, and KIT by TOC‐induced antitumor activity resulted in clinical benefits in 63 of 85 dogs with solid tumors (74%).^3^ However, the mechanism by which TOC serves as postoperative adjuvant treatment for dogs with adenocarcinoma is unclear. If TOC can support improvement in long‐term time to progression (TTP) after surgery in dogs with adenocarcinoma, it could be made widely available for use in this setting.

The presence of microscopic residual disease after surgical removal of tumor tissue is a very important determinant of prognosis in cancer patients. Importantly, recurrence can occur at the primary tumor site even after tumor resection with histopathologically confirmed complete margins.^5^ As previously reported, microscopic tumors can survive and resume growth if the tumor microenvironment (TME) is suitably modulated by angiogenic activity, intratumoral hypoxia, and recruitment of tumor‐infiltrating regulatory T‐cells (Tregs).^6^, ^7^, ^8^ Therefore, therapeutically targeting the TME could prove an effective strategy to prevent relapse or delay tumor growth after surgery.

Sunitinib and sorafenib are 2 multitarget TKIs that have similar therapeutic targets to TOC and are commonly used in human cancer patients. Recent studies have reported that sunitinib and sorafenib could suppress expression of VEGFR2 and hypoxia‐inducible factor (HIF)‐1α as well as decrease the number of tumor‐infiltrating Tregs in several carcinomas of humans, suggesting that these TKIs might target the TME.^9^, ^10^, ^11^, ^12^, ^13^ Consequently, we hypothesized that TOC also could therapeutically target the TME in dogs with adenocarcinoma, and could help improve long‐term TTP after surgery.

Our aim was to investigate the effects of postoperative adjuvant treatment using TOC. Therefore, we retrospectively compared the TTP of 2 subgroups of dogs diagnosed with various types of adenocarcinomas: those treated by surgery alone and those treated by surgery followed by adjuvant treatment with TOC. We also evaluated how TOC affected the TME in terms of VEGFR2 expression and the number of Foxp3^+^ Tregs and HIF‐1α^+^ cells in these tumors. We hypothesized that TOC would improve clinical outcome by modulating the TME.

## 2 MATERIALS AND METHODS

### 2.1 Medical record review

Medical records from 2 referral veterinary facilities in which the authors worked were searched for dogs that were diagnosed with adenocarcinoma and treated between April 2008 and March 2018. Data collected included signalment; clinical signs; results of physical examinations, clinicopathologic assessments, diagnostic imaging, histopathological tests, and staging; treatment; adverse events (AEs); follow‐up information; response to treatment; and, outcomes. The referring veterinarians, owners or both were contacted for follow‐up when additional details were required.

### 2.2 Patients

**FIGURE 1:**
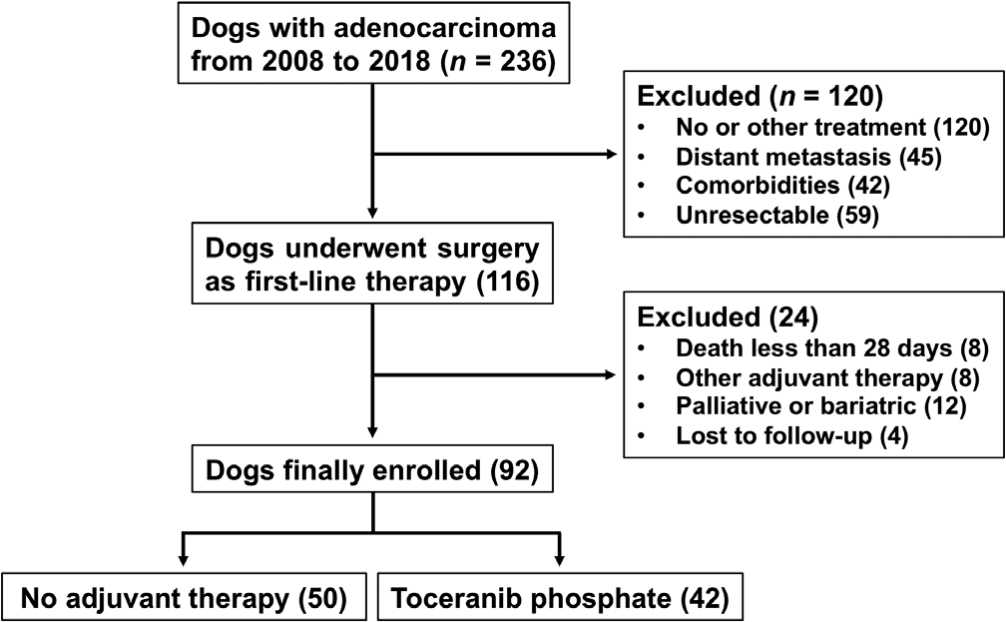
Flow diagram for inclusion and exclusion criteria of the studies

Inclusion criteria used in our study were a histopathological diagnosis of adenocarcinoma. The following clinical information was available for all dogs: history, physical examination findings, clinical signs, hematology, serum biochemistry, urinalysis, and blood pressure measurements. Clinical staging was based on radiography, ultrasonography, or computed tomography. Our study enrolled the dogs that received surgery for a complete remission as first‐line treatment. Cases were excluded from the study under the following circumstances: if surgery was not the primary treatment option, if surgical planning was for surgical debulking or palliative surgery, if other treatments were administered after surgery, if the dogs were affected by distal metastasis, multiple distinct malignant neoplasms or other progressive diseases, and if the follow‐up time was <28 days. A flow diagram illustrating the study's inclusion and exclusion criteria is presented in Figure 1.

### 2.3 Treatment procedures

All dogs underwent excision of the primary tumor, and also the draining lymph node when lymphadenopathy was detected on imaging or as an intraoperative finding. In all dogs diagnosed with solitary lung tumors, lobectomy was performed. For dogs receiving postoperative adjuvant treatment, TOC (Palladia, Zoetis, Florham Park, New Jersey) was administered PO for 21 days after surgery. The dose was calculated to fall within a dosage range of 2.4‐2.9 mg/kg, and was administered every Monday, Wednesday, and Friday.^14^, ^15^ Certain additional medications were allowed to be administered concurrently, including gastroprotectants (H_2_ receptor antagonists or proton pump inhibitors), antiemetics, and antidiarrheals as required. Assessment of AEs was performed every 4 weeks during administration of TOC, in accordance with the veterinary cooperative oncology group–common terminology criteria for AEs.^16^ Adverse events were noted on a graded scale of 1 (mild) to 5 (severe) in the medical records. Treatment with TOC was continued until either disease progression was identified, unacceptable toxicity was reached, or the owner requested that the treatment be discontinued. Dogs were excluded from the study if treatment failure occurred <28 days after first administration, because it is difficult to evaluate the effect of TOC in this context, as previously reported.^17^ The responses and clinical outcomes documented in the medical records were used to determine TTP. This information included records of regular follow‐up communications with the owners and referring veterinarians. Time to progression was defined as the period of time between tumor excision and the point at which local recurrence or metastatic disease was documented.

### 2.4 Western blot analysis

When tumor recurrence after surgery occurred at approximately the same site as the primary location, the dogs underwent a second operation conducted by the same surgeon with the owner's approval. These tumors were histopathologically confirmed as recurrence of the primary tumor. Each pair of tumor specimens collected at the first and second excisions were used for molecular biological analysis as described below.

Vascular endothelial growth factor receptor‐2 protein expression in the tumor samples was evaluated using Western blot analysis. Samples were fragmented using a scalpel, immediately lysed with 300 μL of radioimmunoprecipitation (RIPA) buffer, and then vortexed and homogenized. After homogenization, the insoluble material was removed by centrifugation at 16 000 rpm, and the supernatant was collected for protein quantification. The protein concentrations were determined using the Bradford protein assay in which bovine serum albumin (BSA) was used as the standard. Twenty micrograms of protein from each sample then was loaded onto a gel for electrophoresis. The proteins were denatured, subjected to sodium dodecyl sulfate polyacrylamide gel electrophoresis, and electrotransferred onto nitrocellulose membranes (Whatman, Piscataway, New Jersey) in a semidry transfer apparatus (Bio Craft, Tokyo, Japan). The membranes were incubated for 1 hour at room temperature in a blocking solution: 10 mM Tris‐HCl (pH 7.4), 0.15 M NaCl, 0.1% Tween 20, 1% BSA, and 0.05% NaN_3_. The membranes then were incubated overnight at 4°C with the primary antibodies, including anti‐β‐actin mouse monoclonal antibody (G043; Abcam, Cambridge, Massachusetts) and anti‐mouse VEGFR2 (Flk‐1‐A/3‐: sc‐6251, Santa Cruz Biotechnology Inc, Santa Cruz, California), and diluted 1 : 100 in phosphate‐buffered saline (PBS).^18^ The membranes then were washed 3 times for 5 minutes with Tween‐Tris‐buffered saline (TBS; 10 mM Tris‐HCl, 0.15 M NaCl, and 0.1% Tween 20), and then incubated with a horseradish‐peroxidase‐conjugated anti‐rabbit‐IgG secondary antibody (Fischer Scientific Thermo, Pittsburgh, Pennsylvania) in Tween‐TBS for 1 hour at room temperature. The immunoreactive bands were visualized using a chemiluminescence system (Ez‐Capture MG, Atto, Tokyo, Japan), and detection reagent (Amersham ECL Prime Western Blotting Detection Reagent, GE Healthcare, Princeton, New Jersey). Bands were quantified using quantification software (ImageJ, US National Institutes of Health, version 1.451 http://imagej.nih.gov/ij/, Bethesda, Maryland) and were presented as relative intensities normalized to that of β‐actin.

### 2.5 Immunohistochemistry

Expression of HIF‐1α and Foxp3 proteins in the tumor samples was evaluated using immunohistochemistry (IHC). Sections of the collected tumor samples embedded in paraffin wax blocks were prepared on slides. Samples were subjected to a hydration process by soaking in baths ranging from xylene to graded alcohol, and then washed in PBS (pH 7.2). Next, the slides were fixed in methanol for 15 seconds at room temperature and then air dried. Slides then were immersed in 0.3% H_2_O_2_ in methanol for 20 minutes at room temperature to inhibit endogenous peroxidases, and then washed once more in PBS. Blocking with 10% normal goat serum (Histofine SAB‐PO Kits; Nichirei Bioscience, Tokyo, Japan) then was carried out for 10 minutes, after which the slides were washed 3 times in PBS. The samples were incubated overnight at 4°C with either primary rabbit polyclonal anti‐HIF‐1α antibody (NB100‐449; Novus Biologicals, Littleton, Colorado) diluted 1 : 100 in PBS, or anti‐mouse Foxp3 rat monoclonal antibody (clone FJK‐16 seconds; eBioscience, San Diego, California) diluted 1 : 300 in PBS, washed 3 times with PBS, and then incubated with secondary goat anti‐rabbit IgG (Nichirei Bioscience, Tokyo, Japan) for 30 minutes at 4°C. After 3 more washes with PBS, the samples were incubated in peroxidase streptavidin (Nichirei Bioscience, Tokyo, Japan) for 30 minutes at 4°C, washed 3 times with PBS, and then visualized after staining with 3,3′‐diaminobenzidine (Sigma‐Aldrich, Sigma, St. Louis, Missouri) and hematoxylin. The HIF‐1α^+^ cells were classified as tumor cells in which the nucleus, cytoplasm or both was distinctively stained for HIF‐1α.^19^, ^20^ Regulatory T‐cells were defined as cells with typical lymphocyte morphology and Foxp3 staining in the nucleus but not the cytoplasm.^21^ Regulatory T‐cells were quantified in 2 different compartments: the intra‐tumoral area (defined as the area within nests composed of >5 tumor cells) and the peritumoral area (the area outside tumor cell nests), as reported in a previous study.^21^ Ten high power fields (HPFs) were selected in sites containing >50% tumor cells, and the intratumoral and peritumoral Tregs were counted in each field. Finally, the average Treg number per HPF was calculated.

### 2.6 Statistical analyses

The dogs were divided into 2 subgroups those treated by surgery alone and those treated by surgery followed by adjuvant treatment with TOC. Descriptive and comparative statistics were performed for the overall study population as well as the 2 subgroups. Fisher's exact test was used to compare categorical nonnumerical data such as sex, type of tumor, clinical signs, metastasis, and paraneoplastic syndromes between the 2 subgroups. The Wilcoxon rank‐sum test was used to compare continuous numerical data such as age, weight, and primary tumor size. Kaplan‐Meier survival curves were estimated for the comparison of both clinical outcomes, along with median TTP and 95% confidence intervals (CI). Time to progression was defined as the time period (in days) from the date of surgery until the date of confirmed disease progression (either local recurrence or metastatic disease), and was compared between the 2 subgroups using the log‐rank test. Right‐censored cases were defined as those that showed no relapse or metastasis at the follow‐up closing date (at 1095 days) or those that were lost to follow‐up, died from other causes, or were affected by other diseases or events. Variables analyzed included different types of tumors, signalment, histopathological findings, AEs, and treatment protocol. These variables were evaluated using Cox proportional hazard models. Quantitative values are expressed as means ± SDs from 3 separate experiments. Comparisons of the expression of VEGFR2 protein, the number of intratumoral HIF‐1α^+^ cells and Tregs were performed using the Kruskal‐Wallis test. Results were considered significant when *P* < .05. All statistical analyses were performed using commercial software packages (IBM SPSS version 20.0 IBM Corp., Armonk, New York).

## 3 RESULTS

### 3.1 Patient demographics

Ninety‐two dogs diagnosed with adenocarcinoma fulfilled the study's inclusion criteria, and 18 different breeds were represented in the study population. Of these dogs, 50 (54.3%) were treated by surgery alone, whereas 42 (45.7%) were treated by surgery followed by adjuvant treatment with TOC. The demographic characteristics of the 2 subgroups are shown in Table 1. The types of adenocarcinoma represented in the study population included apocrine gland anal sac adenocarcinoma (AGASA; n = 28), small bowel adenocarcinoma (SBA; n = 24), lung adenocarcinoma (LA; n = 22), and renal cell carcinoma (RCC; n = 18). No significant differences in demographic characteristics were found between the 2 subgroups (Table 1).

**TABLE 1:** Comparison of demographics of the population of dogs with adenocarcinoma classified into two groups: group that received surgery alone and group that received both surgery and toceranib phosphate (TOC)

Variables	Surgery alone (n = 50)	Surgery + TOC^a^ (n = 42)	*P* value
Age (year): median (range)	9.7 (6.8‐14.3)	9.2 (6.2‐12.9)	.94
Weight (kg): median (range)	7.8 (2.4‐28.5)	8.2 (3.2‐36.2)	.99
Sex (n)			.92
Male			
Intact	10 (20%)	9 (21.4%)	
Castrated	16 (32%)	15 (35.7%)	
Female			
Intact	9 (18%)	7 (16.7%)	
Spayed	15 (30%)	11 (26.2%)	
Number of cases			.68
AGASA	16 (32%)	12 (28.6%)	
SBA	12 (24%)	12 (28.6%)	
LA	12 (24%)	10 (23.8%)	
RCC	10 (20%)	8 (19%)	
Area (cm^2^)^b^: median (range)			.82
AGASA	6.4 (0.8‐56)	7.2 (1.2‐48)	
SBA	13.6 (2.8‐135)	15.5 (3.3‐124)	
LA	12.8 (4.2‐56)	16.4 (3.6‐47)	
RCC	19.2 (6.0‐92)	24.2 (6.8‐80)	
Metastasis to LN (n)			.55
AGASA	3 (18.8%)	3 (25%)	
SBA	2 (16.7%)	3 (25%)	
LA	2 (16.7%)	1 (10%)	
RCC	3 (30%)	2 (25%)	

Abbreviations: AGASA, apocrine gland anal sac adenocarcinoma; LA, lung adenocarcinoma; LN, regional lymph node; RCC, renal cell carcinoma; SBA, small bowel adenocarcinoma.

a Surgery + TOC presented treatment with toceranib phosphate within 21 days after surgery.

b Tumor area (cm^2^) at the first examination = (maximum tumor diameter) × (maximum tumor cross‐section diameter).

### 3.2 Adverse events

The majority of dogs receiving TOC tolerated the drug well, but a few instances of severe toxicity were observed. Adverse events were documented in 18 of the 42 dogs receiving TOC (42.9%). Categories, grades, total number, and rate of AEs are presented in Table 2. Ten (55.6%) of the 18 dogs with AEs were given concomitant medications to treat hematological and gastrointestinal events. Ten (55.6%) of the 18 dogs required the TOC dosage to be decreased to <2.4 mg/kg, delayed, or otherwise adjusted, and 5 dogs (27.8%) had the drug withdrawn. Causes for drug withdrawal included gastrointestinal signs, hematological changes, constitutional signs, or a combination of signs. All 42 dogs receiving TOC finally discontinued TOC treatment in <1 year because of AEs (n = 5), tumor relapse or metastasis (18), other diseases (3), or at the owner's request (16). The median administration period for the 42 dogs was 124 days (range, 32‐364).

**TABLE 2:** Adverse events occurring in the dogs receiving toceranib phosphate (TOC)

Categories	Term	Grades					Incidence rate (% of all AEs)
		1	2	3	4	5	
Hematologic	Neutropenia	3	1				18.4%
	Anemia	2					
	Thrombocytopenia	1			1		
	Total	6	1		1		
Metabolic	Increased ALP	2		1			16.2%
	Increased ALT		1				
	Increased AST	1					
	Increased TBili	1					
	Increased BUN		1				
	Hyperglycemia	1					
	Increased CK	1					
	Increased globulin	1					
	Total	7	2	1			
Gastrointestinal	Anorexia	2	1				22.8%
	Vomiting	1	1				
	Nausea	1					
	GI ulceration	1					
	Diarrhea	2			1		
	Hematochezia	1		1			
	Total	8	2	1	1		
Constitutional	Lethargy	1					8.5%
	Weight loss	1					
	Fever	1	1				
	Total	3	1				
Miscellaneous	Proteinuria	1					12.3%
	Cough	1					
	Hypertension	1	1				
	Bilateral tarsal effusion		1				
	Lameness	1		1			
	Motor neuropathy		1				
	Total	4	3	1			
Grand total		28	9	3	2		42.9%

Abbreviation: AEs, adverse events.

### 3.3 Clinical outcomes

In all dogs, histopathologically complete excision was achieved at the initial surgery for the primary tumor. In 35 of the 50 dogs treated by surgery alone, the regional lymph nodes were removed, and metastasis was detected in 10 of these dogs. In 30 of the 42 dogs treated by surgery and TOC, the regional lymph nodes were removed, and metastasis was detected in 9 of these dogs (Table 3). Tumor recurrence, metastasis or both eventually occurred in 60 of the 92 dogs within 1095 days of follow‐up, and 18 of the 60 dogs underwent repeat surgery with the owner's approval. Eight of these 18 dogs (AGASA, n = 3; SBA, 2; LA, 3) had received surgery and TOC and 10 (AGASA, n = 4; SBA, 3; LA, 3) had received surgery alone. Fifteen of the 92 dogs were right‐censored from the clinical research. Ten of these 15 dogs showed no relapse or metastasis at 1095 days, 3 dogs died from other causes, and 2 dogs were lost to follow‐up. Four of the 10 dogs that showed no relapse or metastasis had been treated by surgery alone, and 6 had been treated by surgery and TOC.

**TABLE 3:** Comparison of TTP between subgroups of dogs with adenocarcinoma that received surgery alone

Variables (n)	TTP (days): median (range)				*P* value
	n	Surgery alone	n	Surgery + TOC	
Total cases (92)	50	298 (32‐1095)	42	360 (36‐1095)	.02
Different types of tumors					
Apocrine gland anal sac adenocarcinoma (28)	16	365 (61‐1095)	12	342 (72‐1095)	.18
Small bowel adenocarcinoma (24)	12	302 (64‐1095)	12	380 (75‐1095)	.02
Lung adenocarcinoma (22)	12	145 (47‐886)	10	191 (39‐1095)	.03
Renal cell carcinoma (18)	10	182 (40‐695)	8	256 (48‐836)	.04
Histopathological evaluation					
Invasive type^a^					
Clear (56)	32	328 (40‐1095)	24	365 (48‐1095)	.06
Unclear (36)	20	240 (32‐886)	16	312 (36–1095)	.03
Mitotic index					
≤20/10HPF (48)	26	336 (40–1095)	22	378 (55‐1095)	.16
>20/10HPF (44)	25	223 (32‐756)	19	302 (36‐998)	.01
Vascular invasion					
Positive (32)	20	302 (32–1095)	12	312 (36‐1095)	.62
Negative (60)	32	330 (47‐1095)	28	336 (40‐1095)	.24
Metastasis to LN					
Positive (19)	10	228 (32‐695)	9	267 (36‐886)	.04
Negative (46)	25	360 (61–1095)	21	385 (40–1095)	.36

Abbreviations: HPF, high power field; TOC, toceranib phosphate; TTP, time to progression.

a Invasive type: When border between normal tissue and tumor was unclear, the tumors was defined as invasive type.

**FIGURE 2:**
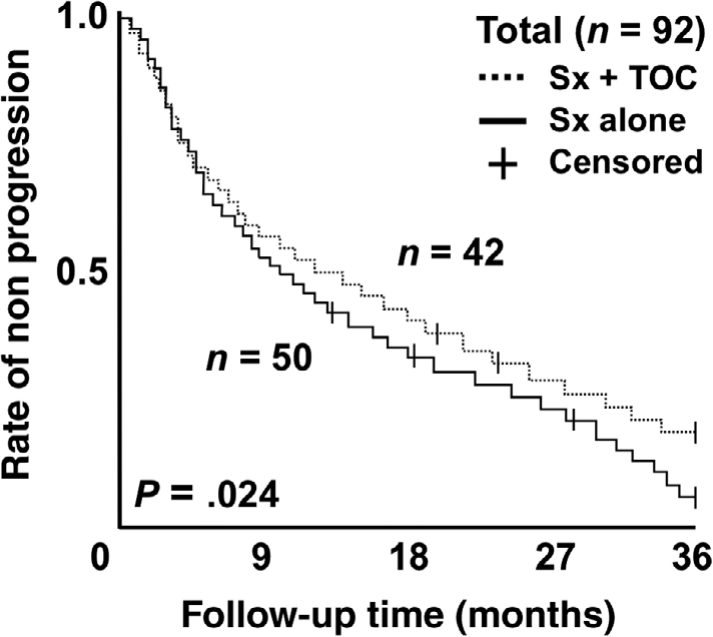
Comparison of time to progression (TTP) between dogs receiving surgery and adjuvant treatment with toceranib phosphate (TOC) and those receiving surgery alone. The hazard ratio of the surgery and TOC group versus the surgery alone group was 0.82 (95% CI, 0.65‐0.96; *P* = .02)

The TTP for the 42 dogs that received surgery and TOC (median, 360 days) was significantly longer than for the 50 dogs that received surgery alone (median, 298 days; hazard ratio [HR], 0.82; 95% CI, 0.65‐0.96; *P* = .02; Figure 2 and Table 3). Comparisons of other variables are shown in Table 3. Among SBA, LA, and RCC cases, the TTP for dogs treated by surgery and TOC was significantly longer than for those treated by surgery alone, but no significant difference in TTP was found between the 2 treatment groups for AGASA cases (Table 3). Among dogs with an invasive type of tumor (ie, >20/10 HPF mitotic index and lymph node metastases), the TTP for dogs treated by surgery and TOC was significantly longer than for those treated by surgery alone (Table 3). In the 42 dogs that received surgery and TOC, no significant differences were found when comparing the incidence of AEs (Table 4). However, a significantly longer TTP was associated with the following variables during TOC treatment: systolic blood pressure > 136 mm Hg (HR, 0.76; 95% CI, 0.59‐0.93; *P* = .04), neutrophil count ≤4200/μL (HR, 0.81; 95% CI, 0.63‐0.97; *P* = .02), total dose >112 mg/kg (HR, 0.72; 95% CI, 0.45‐0.89; *P* = .0082), and administration period >124 days (HR, 0.78; 95% CI, 0.62‐0.91; *P* = .02; Table 4).

**TABLE 4:** Comparison of time to progression (TTP) in the dogs receiving toceranib phosphate (TOC)

Variables (n)	TTP (days) median (range)	*P* value
Adverse events		
Incidence of AEs		
Presence (18)	356 (36‐1095)	.64
Absence (24)	364 (47‐1095)	
Systolic blood^a^ pressure		
≤136 mm Hg (21)	324 (36‐1095)	.04
>136 mm Hg (21)	387 (48‐1095)	
Neutrophil count^b^		
≤4200/μL (21)	398 (47‐1095)	.02
>4200/μL (21)	318 (36‐1095)	
Treatment protocol		
Total dose^c^		
≤112 mg/kg (21)	324 (36‐1095)	.01
>112 mg/kg (21)	398 (47‐1095)	
Administration period		
≤124 days (21)	331 (36‐1095)	.05
>124 days (21)	379 (40‐1095)	

a Systolic blood pressure showed mean value measured during TOC treatment.

b Neutrophil count showed mean value measured during TOC treatment.

c Total dose of TOC which administrated as postoperative adjuvant treatment (dose of 112 mg/kg showed mean value in the dogs receiving TOC).

### 3.4 Molecular biological assessment

**FIGURE 3:**
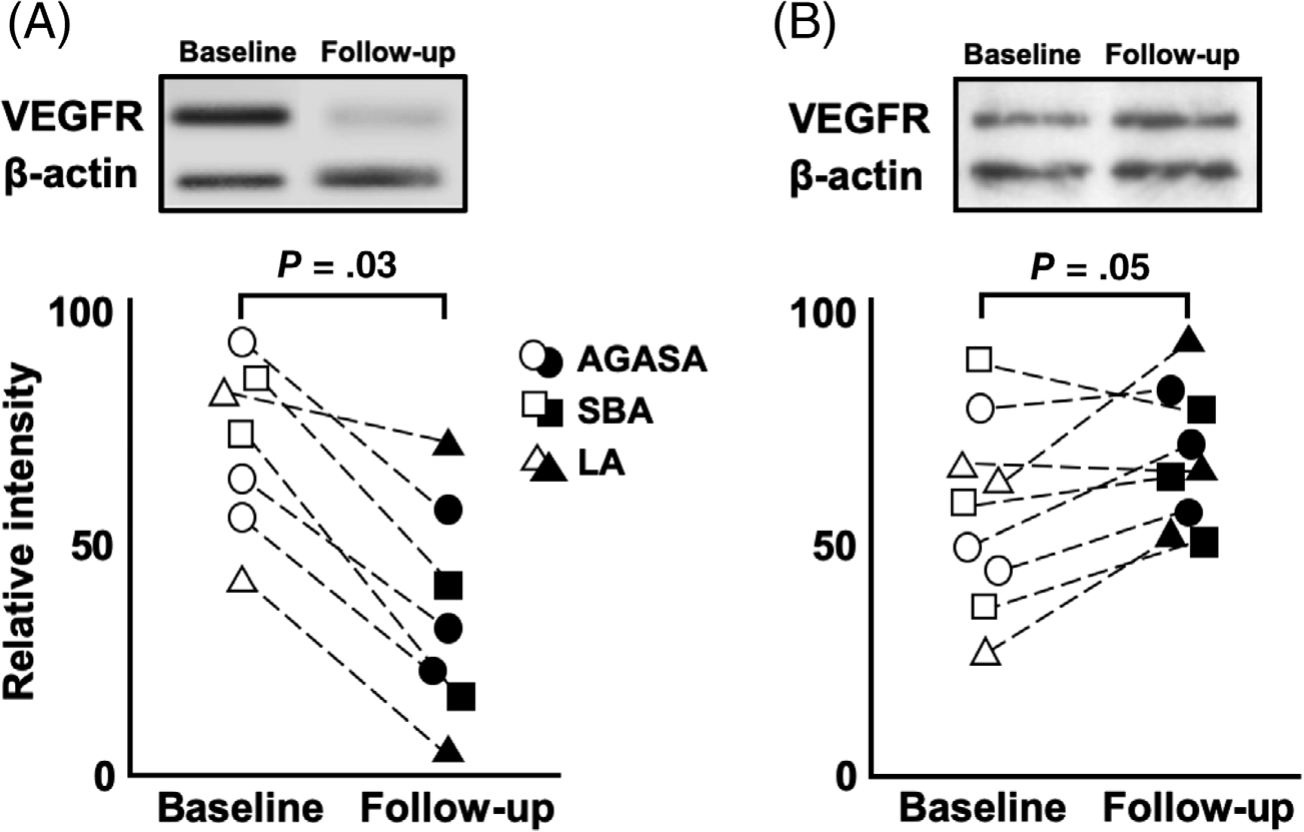
Expression of VEGFR2 was assessed by western blotting analysis in the tumor specimens collected at first and second surgery (Figure 2 showed small bowel adenocarcinoma). In dogs receiving surgery and toceranib phosphate, the relative intensities of the immunoreactive bands was significantly decreased in the second group of specimens (follow‐up) when compared to the first group of specimens (baseline) (*P* = .03; A). In dogs receiving surgery alone, there was no significant difference between baseline and follow‐up (*P* = .05; B)

**FIGURE 4:**
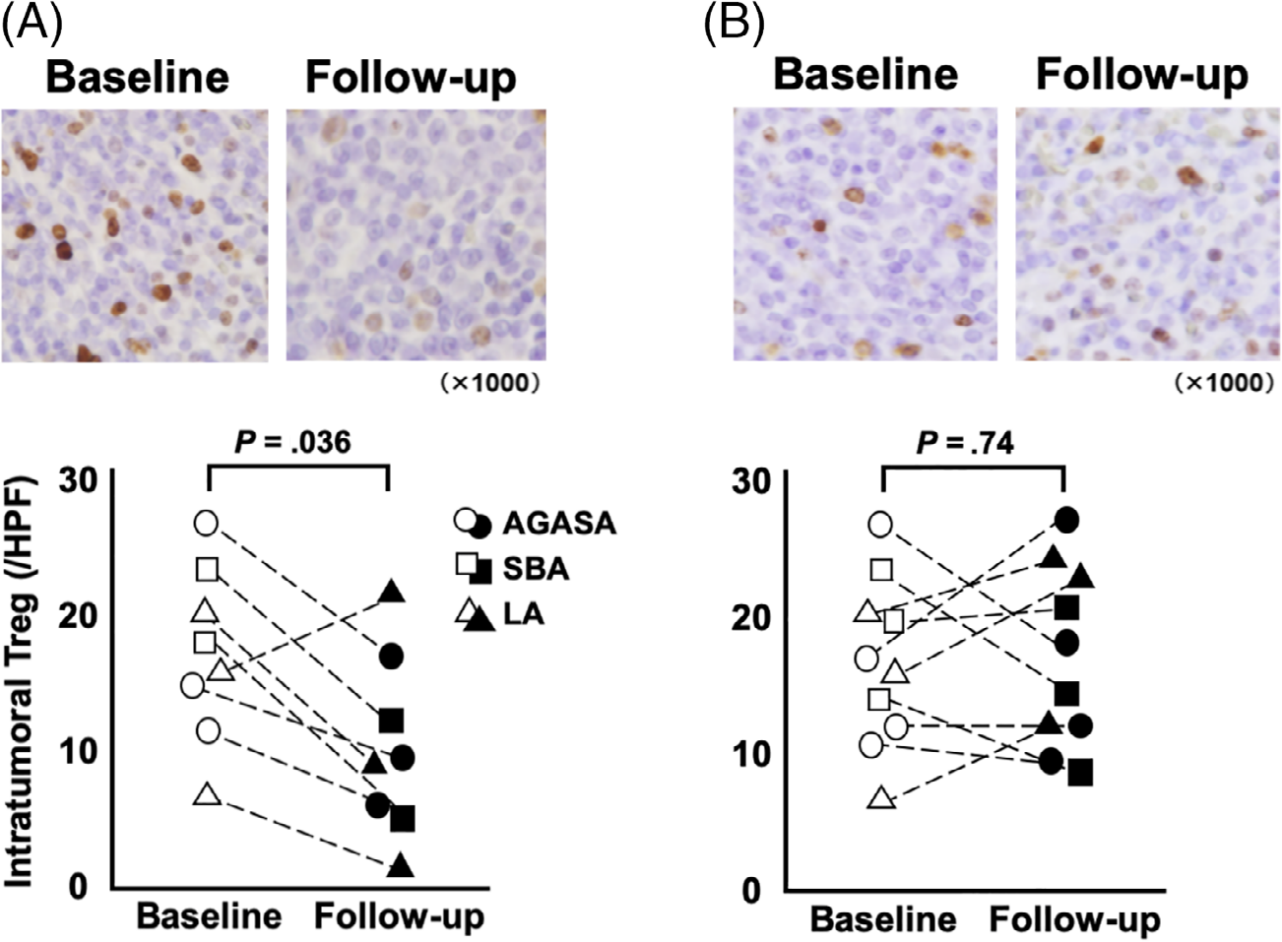
Foxp3^+^ regulatory T‐cells were assessed by immunohistochemistry in the tumor specimens at first and second surgery (Figure 3 showed small bowel adenocarcinoma). In dogs receiving surgery and toceranib phosphate, the median number of Foxp3^+^ Treg at baseline and follow‐up were 18.5 and 11.2 cells per high power field (HPF), respectively, and follow‐up significantly decreased when compared to baseline (*P* = .04, A). In dogs receiving surgery alone, the median of baseline and follow‐up were 16.3 and 17.2 cells per HPF, respectively, and there was no significant between baseline and follow‐up (*P* = .74, B)

**FIGURE 5:**
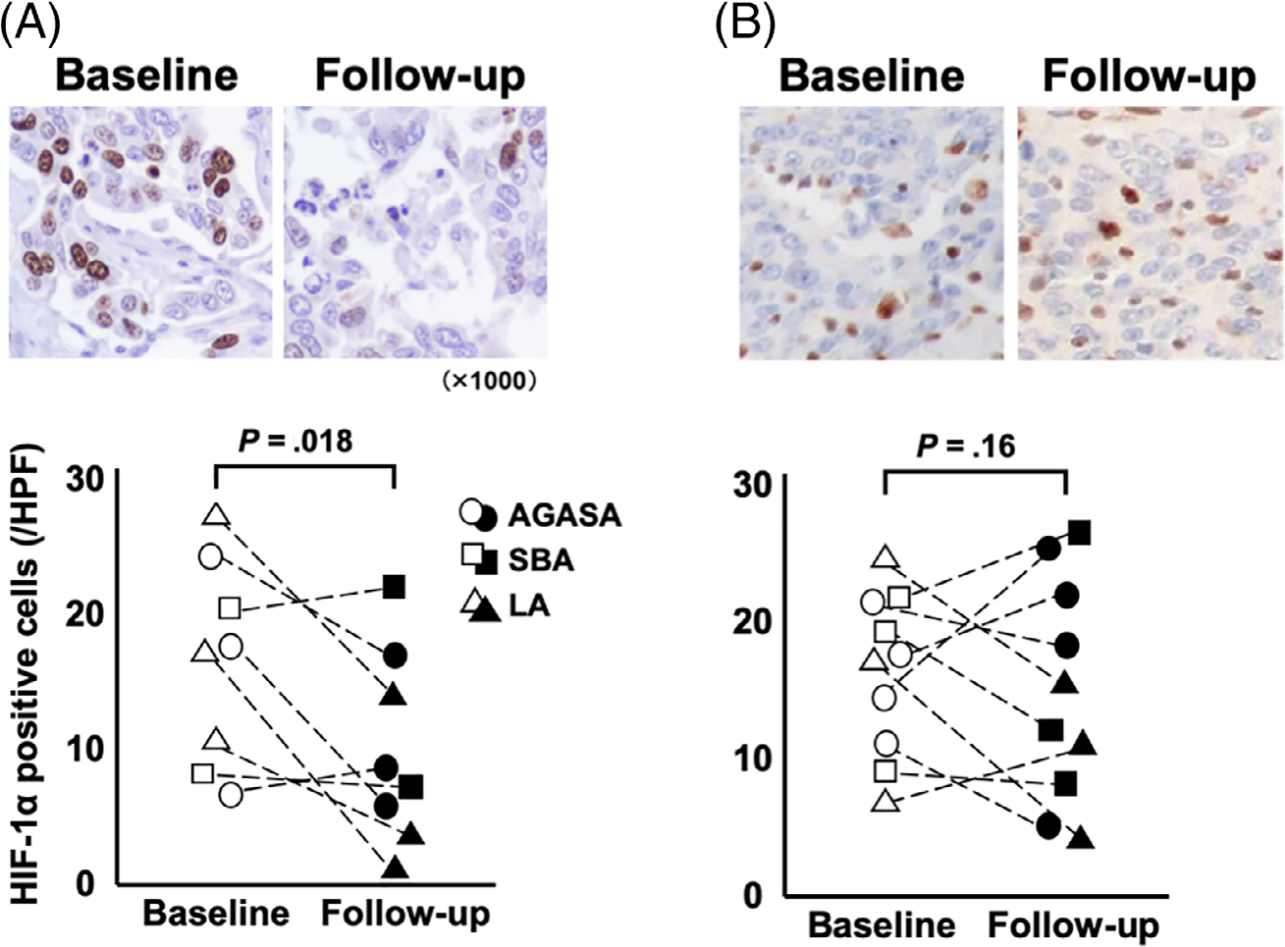
HIF‐1α^+^ cells were assessed by immunohistochemistry in the tumor specimens collected at first and second excision (Figure 4 showed small bowel adenocarcinoma). In dogs receiving surgery and toceranib phosphate, the median number of HIF‐1α^+^ tumor cells at baseline and follow‐up were 17.8 and 8.5 cells per high power field (HPF), respectively; there was a significant decrease at follow‐up when compared to baseline (*P* = .02; A). In dogs receiving surgery alone, the median of baseline and follow‐up were 17.1 and 14.4 cells per HPF, respectively; there was no significant difference between baseline and follow‐up (*P* = .16; B)

Expression of VEGFR2 protein was comparatively evaluated between specimens collected at the first and second surgeries in 16 of the 18 cases that underwent repeat surgery (Figure 3). Expression of HIF‐1α and Foxp3 proteins was comparatively evaluated between specimens taken at the 2 surgeries in all 18 cases (Figures 4 and 5). In dogs that were treated by surgery and TOC, the relative intensities of the immunoreactive bands of VEGFR2 were significantly lower in the second group of specimens (follow‐up) than in the first group of specimens (baseline; *P* = .03; Figure 3A). In dogs that were treated by surgery alone, no significant difference was found in VEGFR2 expression between baseline and follow‐up (*P* = .05; Figure 3B).

Foxp3^+^ Treg were observed in both the intratumoral and peritumoral areas of the specimens (Figure 4). In dogs treated by surgery and TOC, the median number of Foxp3^+^ Treg at baseline and follow‐up was 18.5 and 11.2 cells per HPF, respectively, and this decrease was statistically significant (*P* = .04; Figure 4A). In dogs treated by surgery alone, the median number of Foxp3^+^ Treg at baseline and follow‐up showed no significant difference (16.3 and 17.2 cells per HPF, respectively; *P* = .74; Figure 4B).

The HIF‐1α^+^ cells were observed in tumor tissues (Figure 5). In dogs treated by surgery and TOC, the median numbers of HIF‐1α^+^ tumor cells at baseline and follow‐up were 17.8 and 8.5 cells per HPF, respectively, which represents a significant decrease in the number of these cells at follow‐up when compared to baseline (*P* = .02; Figure 5A). In dogs treated by surgery alone, the median numbers of HIF‐1α^+^ tumor cells at baseline and follow‐up were 17.1 and 14.4 cells per HPF, respectively, which represents no significant difference between these results (*P* = .16; Figure 5B).

## 4 DISCUSSION

Ours is the first study to investigate the efficacy of a single agent, TOC, as a postoperative adjuvant treatment for dogs with adenocarcinoma. We compared the clinical outcomes of 2 subgroups with similar demographic characteristics. In veterinary oncology, survival time often is used as the primary end point because it is easily defined. However, it can be affected by owner‐driven factors such as the delay of initiation of additional treatments or euthanasia. We therefore used TTP, because it is a more reliable method for assessing response to treatment. In our study, adjuvant treatment with TOC was associated with an overall improvement of TTP in dogs with adenocarcinoma. Intriguingly, TOC significantly increased the TTP for dogs suffering from SBA, LA, and RCC, and some advanced types of adenocarcinomas that had higher mitotic index, invasive characteristics, and lymph node metastases. Several studies have reported that postoperative adjuvant treatment with sunitinib and sorafenib was efficacious in human patients with advanced RCC.^22^, ^23^, ^24^ Reports in the veterinary literature also have indicated that postoperative adjuvant treatment may improve survival time in dogs with SBA.^25^ Our data suggest that the effects of TOC might depend on the type of adenocarcinoma in dogs that it is used to treat.

Dogs treated with TOC in general tolerated it well, but these cases had a higher rate of AEs as compared to those treated by surgery alone. These incidence rates are consistent with a previously published study.^26^ Our study suggested that AEs caused by TOC do not necessarily disadvantage the patients. Our data indicated that higher systolic blood pressure and lower neutrophil count were associated with significantly prolonged TTP in dogs that received TOC, whereas dogs treated by surgery alone had lower systolic blood pressure and higher neutrophil count. Consequently, it is important to note that dogs experiencing AEs were effectively managed by dose adjustments, drug holidays, concomitant medications or some combination of these, to avoid early drug withdrawal.

Therapeutic approaches that target the TME are important for the prevention of recurrence and metastasis.^27^ The veterinary literature increasingly has recognized the importance of various aspects of the TME, including angiogenic activity, intratumoral hypoxia, and tumor‐infiltrating Tregs. Indeed, some clinical studies have shown that TOC treatment significantly decreased the number of circulating Tregs, and increased the plasma concentration of VEGF in dogs with tumors.^14^, ^15^ We found that TOC treatment inhibited VEGFR2 expression and decreased the number of HIF‐1α^+^ tumor cells and Foxp3^+^ Tregs within the tumor tissue, although little is known about the inhibitory mechanisms. In support of our findings, it has already been determined that some TKIs suppress HIF‐1α synthesis under hypoxic conditions and inhibit tumor infiltrating Tregs in several carcinomas of humans, suggesting that it might be a principal mechanism of action.^10^, ^11^, ^12^, ^13^ In clinical research in humans, an increase in tumor‐infiltrating Tregs and high expression of HIF‐1α and VEGFR have been reported to be correlated with tumor recurrence or distant metastasis.^6^, ^7^, ^8^

Administration of TOC at a dosage of 2.75 mg/kg every other day (EOD) significantly decreased the number of circulating Tregs in dogs with tumors, possibly by immunomodulatory mechanisms, and TOC treatment at a dosage of 2.4‐2.9 mg/kg EOD increased plasma VEGF concentrations, which is consistent with VEGFR inhibition.^14^, ^15^ In our study, TOC treatment (dose range, 2.4‐2.9 mg/kg, every Monday, Wednesday, and Friday) also had various modulatory effects in the TME, inhibiting VEGFR2 expression and decreasing the number of tumor‐infiltrating Tregs and HIF‐1α^+^ tumor cells. In addition, AEs from this dosage were mainly limited to mild hematological or gastrointestinal events that required minimal supportive care or dose adjustment. Although the administered dosage and frequency were lower than the manufacturer's recommendations (3.25 mg/kg, PO, EOD), this dosage regimen appears to have been clinically useful and safe for dogs with adenocarcinomas.

Our data indicated that TOC significantly increased the TTP for dogs with adenocarcinomas, and decreased the number of intratumoral Foxp3^+^ Tregs and HIF‐1α^+^ tumor cells, as well as inhibited VEGFR2 expression. Our study had several limitations, including its retrospective design, the fact that multiple centers were used, and the small patient population. In each clinical institution, there were some differences in historical background between the groups, and differences in surgical proficiency or time might have affected surgical outcomes to some extent. Most importantly, we could not directly determine whether inhibition of the targets in the TME‐affected TTP. Additionally, some of the dogs that received TOC experienced tumor recurrence despite significant modulation of the TME. Although some veterinary researchers previously have demonstrated how the number of Foxp3^+^ Tregs and the expression of VEGFR2 or HIF‐1α can affect clinical outcomes and therapeutic benefits in dogs with tumors,^19^, ^20^, ^21^, ^28^, ^29^ the relationship between the modulation of expression of these proteins and the mechanism of recurrence remains unclear. One investigator suggested that tumor recurrence might be independent of angiogenesis.^30^ In some dogs with adenocarcinomas, tumor recurrence might not be influenced by Foxp3^+^ Tregs, VEGFR2 or HIF‐1α, and may effectively take advantage of other critical microenvironmental factors. To establish the therapeutic approach of using TOC to target the TME, further prospective research on the relationship between TME modulation and the process of recurrence is required.

In conclusion, we determined that postoperative adjuvant treatment with TOC was associated with a significantly longer TTP for dogs with adenocarcinomas compared to treatment by surgery alone, and that this inhibition of recurrence possibly is caused by the long‐term inhibition of Tregs, VEGFR2, and HIF‐1α associated with TOC treatment. Our data should contribute to developing a prospective therapeutic approach for using TOC to target the TME. Further research is required to establish clinical applications in small animals.

## OFF‐LABEL ANTIMICROBIAL DECLARATION

Authors declare no off‐label use of antimicrobials.

## CONFLICT OF INTEREST DECLARATION

Authors declare no conflict of interest.

## INSTITUTIONAL ANIMAL CARE AND USE COMMITTEE (IACUC) OR OTHER APPROVAL DECLARATION

Authors declare no IACUC or other approval was needed.

## HUMAN ETHICS APPROVAL DECLARATION

Authors declare human ethics approval was not needed for our study.
